# The Harm in Euphoria: Exploring the Benefit of Harm Reduction Strategies in the Narrative of Rue Bennett From ‘Euphoria'

**DOI:** 10.1192/bjo.2024.228

**Published:** 2024-08-01

**Authors:** Iyinoluwa Popoola

**Affiliations:** King's College London, London, United Kingdom

## Abstract

**Aims:**

"Euphoria,” an American television show portraying the lives of teenagers, centers around Rue Bennett, a seventeen-year-old biracial girl grappling with substance misuse and comorbid mental health conditions, including obsessive-compulsive disorder and bipolar disorder. Rue's risky behaviors in the series mirror real-life challenges faced by adolescents dealing with substance misuse. This study aims to explore harm reduction strategies that could benefit Rue, emphasizing the need for such approaches to improve safety in non-abstinent adolescents. By focusing on harm reduction rather than strict abstinence, the goal is to meet individuals where they are in their journey and foster sustainable positive change.

**Methods:**

Employing a qualitative approach, this study conducted a thematic content analysis of relevant episodes from seasons 1 and 2 of “Euphoria.” Additionally, a literature search was carried out using online databases, including PubMed, PsychINFO, and Google Scholar, to identify relevant literature on harm reduction strategies for adolescent opiate users from 2019 to 2024.

**Results:**

The analysis uncovered multiple instances of Rue's risky behavior. Major themes included polydrug use, self-medication, overdose, association with dangerous individuals, self isolation and withdrawal management. Examining Rue's journey identified harm reduction strategies which could minimise her risk of harm, such as fentanyl test strips, Narcan, and psychoeducation in safer consumption practices, supported by existing literature.

**Conclusion:**

Rue Bennett's character in “Euphoria” underscores the imperative need for harm reduction approaches in substance use interventions for adolescents. The study highlights the potential effectiveness of harm reduction strategies, including Narcan and psychoeducation in minimizing risks associated with opiate use. Rue's narrative emphasizes how these methods could contribute to creating a safer consumption environment for non-abstinent individuals. Integrating harm reduction principles into real-world interventions is crucial for promoting holistic well-being and challenging stigmatizing attitudes toward substance use.

